# Control of Cardiac Output with Ivabradine or Beta-Blockers for Refractory Hypoxemia under Veno-Venous ECMO for Severe ARDS

**DOI:** 10.1007/s10557-024-07650-5

**Published:** 2024-12-30

**Authors:** Paul Masi, Lionel Tchatat Wangueu, François Bagate, Alexandra Plesa, Thierry Folliguet, Armand Mekontso Dessap

**Affiliations:** 1 AP-HP, Hôpitaux Universitaires Henri-Mondor, Service de Médecine Intensive Réanimation, 94010 Créteil, France; 2 Univ Paris Est Créteil, CARMAS, 94010 Créteil, France; 3 AP-HP, Hôpital Henri Mondor, Pharmacie, 94000 Créteil, France; 4 Service de Chirurgie Cardiaque, DMU CARE, Assistance Publique-Hôpitaux de Paris (AP-HP), Hôpitaux Universitaires Henri Mondor, 94010 Créteil, France; 5 Université Paris Est Créteil, Faculté de Santé, 94010 Créteil, France; 6 Service de Réanimation Médicale, Hôpital Henri Mondor, Créteil, France

**Keywords:** Refractory hypoxemia, Veno-venous ECMO, Ivabradine, Beta blockers

## Abstract

**Purpose:**

Hypoxemia is a risk factor for mortality and long-term neuropsychological impairment during severe acute respiratory distress syndrome (ARDS). Veno-venous extracorporeal membrane oxygenation (VV-ECMO) is a potential treatment for such cases but may not suffice. We aimed to evaluate the effects of pharmacological interventions for cardiac output (CO) control using ivabradine or beta-blockers for refractory hypoxemia during VV-ECMO.

**Methods:**

The study involved retrospective analysis of consecutive patients with severe ARDS who underwent VV-ECMO at a tertiary university hospital between March 2020 and May 2022. Patients with refractory hypoxemia under VV-ECMO were included. Pharmacological interventions included ivabradine and/or short half-life beta-blockers. The primary endpoint was the change in ECMO flow/CO ratio and secondary endpoints were changes in macrocirculation (mean arterial pressure), oxygenation [arterial saturation (SaO_2_) and oxygen transport (DO_2_)] and tissue hypoxia (lactate levels).

**Results:**

Out of 70 patients on VV-ECMO, ten had refractory hypoxemia under VV-ECMO and received pharmacological interventions to control CO. The ECMO flow/CO ratio significantly increased with pharmacological intervention overall (from 60% [50–66] to 69% [61–81], *p* = 0.02), as well as with beta-blockers or ivabradine individually. However, DO_2_ decreased, especially with beta-blockers and to some extent with ivabradine. There were no reported immediate adverse events, and lactate levels remained below the anaerobic threshold.

**Conclusion:**

Ivabradine and beta-blockers were clinically well-tolerated and improved the ECMO flow/CO ratio in patients with refractory hypoxemia during VV-ECMO. However, the improvement of arterial oxygenation was associated with decreased DO_2_.

**Supplementary Information:**

The online version contains supplementary material available at 10.1007/s10557-024-07650-5.

## Background

In severe acute respiratory distress syndrome (ARDS), hypoxemia is a risk factor for mortality and long-term neuropsychological impairment [1]. Veno-venous extracorporeal membrane oxygenation (VV-ECMO) can be used for refractory hypoxemia in this setting [2]. For most patients in this situation, ECMO blood flow ensures adequate oxygenation. In addition, prone positioning can be attempted to optimize residual lung function, but it does not improve survival in COVID-19-related ARDS receiving VV-ECMO [3]. If persistent hypoxemia occurs during the ECMO course and recirculation, cannula positioning and membrane function issues have been ruled out, increasing ECMO flow is recommended to the maximum extent possible without causing excessive hemolysis [4]. An ECMO flow to cardiac output (CO) ratio greater than 60% is associated with better blood oxygenation, oxygen transport, and oxygen delivery (DO_2_) [5]. Some teams and experts suggest using beta blockers to optimize ECMO flow/CO ratio, as it is associated with an increase in arterial oxygen saturation (SaO_2_) [6, 7], especially when patients present with high cardiac output, defined as a cardiac index (CI) > 4 L/min/m^2^ [8]. However, their effects on hemodynamics and oxygen transport are not well characterized in this indication. In addition, the role of other bradycardic agents with pure chronotropic effects like ivabradine has not been assessed in this setting. In our center, we have implemented a protocol of CO control with beta blockers or ivabradine for refractory hypoxemia under VV-ECMO. The present study was conducted to comprehensively evaluate this strategy in terms of macrocirculation, oxygenation, and tissue hypoxia.

## Methods

Consecutive patients who required VV-ECMO between March 2020 and May 2022 in the medical intensive care unit (ICU) of a tertiary university hospital, which also hosts a mobile circulatory assistance unit, were retrospectively assessed for eligibility. Patients with refractory hypoxemia despite maximal support were included, defined as having an SaO_2_ < 88% despite optimizing ECMO flow (i.e., to the highest level not inducing overt hemolysis) and excluding recirculation. Patients were also included if SaO_2_ was < 92% with signs of hemolysis precluding a further increase in ECMO blood flow, i.e., plasma free hemoglobin > 50 mg/mL and/or lactate dehydrogenase > 600 IU/L. Recirculation was suspected if the blood in the inflow cannula had an oxygen saturated color, or if the distance between the two cannulas was small (< 10 cm). Hemolysis and membrane lung dysfunction were assessed at least daily by checking the visual appearance of the membrane and obtaining post-membrane blood gases if membrane dysfunction was suspected.

CO was measured using echocardiography, and various biological (i.e., serum lactate dehydrogenase, fibrinogen, free plasma hemoglobin, carboxyhemoglobin and platelet counts) and clinical parameters were collected. The first step of CO control involved achieving moderate hypothermia with a target temperature of 35–36 °C. Subsequently, ivabradine was introduced for patients in sinus rhythm, at a dose of 7.5 mg twice a day. If refractory hypoxemia persisted despite these interventions, ivabradine was discontinued, and a short half-life beta-blocker was administered, using landiolol (started at 1 µg/kg/min, and increased up to 40 µg/kg/min) or esmolol (started at 50 µg/kg/min and increased up to 300 µg/kg/min). Hemodynamic parameters were assessed before and after hemodynamic intervention.

Statistical analyses were performed with the JMP software (version 9; SAS Institute Inc, Cary, NC) and GraphPad Prism software (version 5; GraphPad Software Inc., La Jolla, CA, USA). The primary endpoint of this study was the change in ECMO flow/CO ratio induced by the pharmacological intervention. The secondary endpoints included the effects on macrocirculation (as assessed by mean arterial pressure), oxygenation (as assessed by SaO_2_ and DO_2_), and tissue hypoxia (as assessed by lactate levels). Data are presented as median with interquartile range or number with percentage. Multiple paired values were compared using Wilcoxon test. For all tests, a two-way *p* value < 0.05 was considered statistically significant.

## Results

During the study period, a total of 70 patients required VV-ECMO for severe ARDS, including 60 without refractory hypoxemia (21 deaths, 35%) and ten with refractory hypoxemia (eight deaths, 80%) (Fig. S1). The ten patients with refractory hypoxemia had a median age of 48 [33–58] years, and a median ECMO duration and length of stay in the ICU of 59 [36–92] and 81 [37–120] days, respectively (Table S1).

Overall, a total of 17 pharmacological interventions were performed in these patients, nine with ivabradine and eight with beta-blockers. Before intervention, median SpO_2_ was low (88 [82.5–90]), despite optimization of ECMO flow (5.5 [4.4–6.2] L/min, with admission and reinjection cannula of relatively large size of 26 [25–27] and 21 [19–21] F, respectively), and other interventions, including red blood cell transfusion in 15 (88%) cases, prone position in nine (53%) cases and oxygenator membrane change in five (29%) cases (Table S2). The distance between the two cannulas (16 [14–17] cm) suggested no or minimal recirculation. Before intervention, most patients also experienced bleeding and significant hemolysis, with lacticodeshydrogenase of 719 [518–925] U/L, free plasmatic hemoglobin of 110 [52–202] mg/L, and carboxyhemoglobin of 2.9 [1.6–3.4]% (Table S2).

**Fig. 1:**
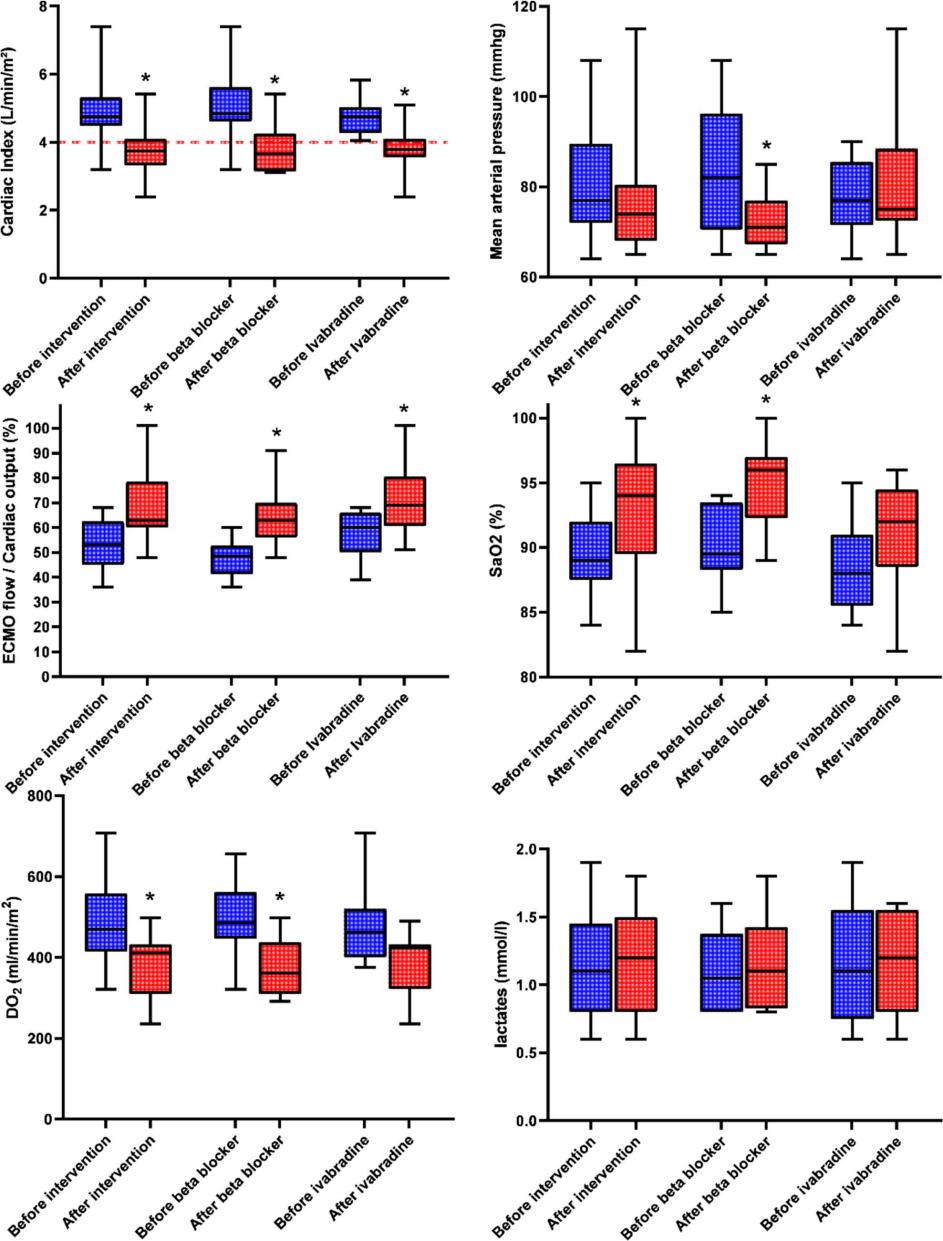
Box and whiskers plots of various hemodynamic parameters before and after pharmacological intervention with ivabradine or beta-blocker for refractory hypoxemia, including cardiac index (dotted line represent a CI > 4 L/min/m^2^), mean arterial pressure, the ratio of extracorporeal membrane oxygenation flow to cardiac output, arterial oxygen saturation, oxygen delivery, and arterial lactate. *p value < 0.05 for paired Wilcoxon test

The effects of pharmacological interventions are reported in Table 1 and Fig. 1, with comparison of physiological variables before and after intervention. The median delay between the two assessments was 2 [1–3] days. No immediate adverse event was reported, in particular severe bradycardia (< 50 bpm) or cardiac arrest. The ECMO flow/CO significantly increased with pharmacological intervention overall (from 60% [50–66] to 69% [61–81], *p* = 0.02), as well as with beta-blockers and ivabradine individually. In terms of CI, almost all patients experienced a high cardiac output state before the intervention, as shown in Fig. 1. After the intervention, a significant portion of them had a CI below 4 L/min/m^2^, with no impact on lactate levels. Mean arterial pressure significantly decreased with beta-blockers but not with ivabradine. SaO_2_ significantly increased while DO_2_ significantly decreased overall and with beta-blockers; these trends did not achieve statistical significance with ivabradine. Lactate levels remained below the anaerobic threshold in all situations. After 3 years of follow-up, only two patients survived. The first required long-term oxygen therapy until 10 months after hospital discharge, while the second was discharged from the hospital without oxygen support but experienced persistent muscle weakness for 6 months.

**Table 1:** Effect pharmacological intervention for refractory hypoxemia on hemodynamics, oxygenation, and tissue perfusion

Variables	Before intervention	After intervention	*P* value
All cohort (*n* = 17)			
ECMO flow/cardiac output ratio (%)	53 [45–63]	63 [60–79]	< 0.0001
ECMO flow (L/min)	5.5 [4.4–6.2]	5.5 [4.2–6.2]	0.9
Cardiac output (L/min)	10.1 [9.2–11.1]	7.9 [7–8.7]	0.0002
Cardiac index (L/min/m^2^)	4.7 [4.5–5.3]	3.7 [3.3–4.1]	< 0.0001
High cardiac index* (%)	16 (94)	6 (35)	0.0039
Temperature (°C)	36.2 [35.5–36.6]	35.5 [35–36]	0.05
Mean arterial pressure (mmHg)	77 [72–90]	74 [68–81]	0.25
Lactates (mmol/L)	1.1 [0.8–1.5]	1.2 [0.8–1.5]	0.93
Hemoglobin (g/dL)	8.4 [8–8.6]	8.2 [7.8–8.6]	0.3
SaO_2_ (%)	89 [88–92]	94 [90–97]	0.006
PaO_2_ (mmHg)	57 [53–63]	62 [57–74]	0.06
DO_2_ (mL/min/m^2^)	469 [414–558]	411 [309–433]	0.001
Ivabradin (*n* = 9)			
ECMO flow/cardiac output ratio (%)	60 [50–66]	69 [61–81]	0.02
ECMO flow (L/min)	6 [5–6.4]	6 [4.4–6.6]	0.66
Cardiac output (L/min)	9.8 [9.2–10.8]	8 [7.3–8.9]	0.02
Cardiac index (L/min/m^2^)	4.7 [4.3–5]	3.8 [3.6–4.1]	0.019
High cardiac output* (%)	9 (100)	4 (44)	0.03
Temperature (°C)	36.5 [36.3–36.6]	35.4 [34.9–36.7]	0.08
Mean arterial pressure (mmHg)	77 [72–86]	75 [73–89]	0.71
Lactates (mmol/L)	1.1 [0.8–1.6]	1.2 [0.8–1.6]	0.68
Hemoglobin (g/dL)	8.4 [7.8–8.7]	8.5 [8–9, ]	0.87
SaO_2_ (%)	88 [86–91]	92 [89–95]	0.14
PaO_2_ (mmHg)	55 [51–63]	60 [55–62]	0.62
DO_2_ (mL/min/m^2^)	463 [400–520]	424 [321–433]	0.16
Beta blocker (*n* = 8)			
ECMO flow /cardiac output ratio (%)	49 [41–53]	63 [56–70]	0.001
ECMO flow (L/min)	5.2 [4.1–6]	5.3 [4.2–5.5]	0.62
Cardiac output (L/min)	10.6 [9.5–11.2]	7.8 [6.6–8.6]	0.016
Cardiac index (L/min/m^2^)	4.8 [4.6–5.6]	3.7 [3.1–4.3]	0.0005
High cardiac output* (%)	7 (88)	2 (25)	0.25
Temperature (°C)	35.5 [35–36]	35.5 [35.1–35.8]	0.48
Mean arterial pressure (mmHg)	82 [71–96]	71 [67–77]	0.02
Lactates (mmol/L)	1 [0.8–1.4]	1.1 [0.8–1.4]	0.73
Hemoglobin (g/dL)	8.4 [8.1–8.6]	8 [7.8–8.5]	0.12
SaO_2_ (%)	90 [88–94]	96 [92–97]	0.04
PaO_2_ (mmHg)	58 [53–66]	71 [60–81]	0.16
DO_2_ (mL/min/m^2^)	486 [446–562]	361 [309–438]	0.008

*ECMO* extracorporeal membrane oxygenation, *DO*_*2*_ oxygen delivery

^*^defined by a cardiac index ≥ 4 L/min/m^2^

## Discussion

The present study represents the first attempt to comprehensively investigate the effects of both ivabradine and short half-life beta-blockers in managing refractory hypoxemia during VV-ECMO. Our findings indicate that these pharmacological interventions may both improve the ECMO flow/CO ratio and arterial oxygenation but with a reduction in DO_2_, particularly with beta-blockers.

It is noteworthy that mortality is very high in our cohort, with most deaths being due to multi-organ failure and severe hemorrhagic complications, particularly cerebral. These seem unrelated to the bradycardic treatment. Only two patients who experienced refractory hypoxemia while on ECMO survived during the 3 years of follow-up.

The balance between increasing arterial saturation and preserving net oxygen delivery is physiologically critical. A minor increase in arterial saturation, achieved by reducing CO, might not always translate into improved tissue oxygenation, given the potential decrease in DO_2_. Consequently, continuous monitoring of CO, oxygen consumption, and delivery is essential in managing refractory hypoxemia under VV-ECMO. We observed a significant reduction in DO_2_, especially with beta-blockers, in accordance with previous reports [6, 7]. While there was no evidence of tissue hypoxia, it is essential to be cautious about reaching the anaerobic threshold defined by lactate production [9].

Hemoglobin levels lower than 7 g/dL under VV-ECMO have been associated with increased mortality [10]. Transfusion to target a higher hemoglobin level is an interesting option to optimize DO_2_ but comes with potential side effects, such as increased risk of transfusion-related acute lung injury (TRALI) [11] and transfusion-associated circulatory overload (TACO) [12]. Achieving higher targets of hemoglobin level to improve DO_2_ may be difficult in practice given the frequent hemorrhagic complications and hemolytic burden under VV-ECMO, as exhibited by our patients [13].

Managing refractory hypoxemia remains challenging and requires a multi-faceted approach. Monitoring hemolysis parameters and ensuring proper membrane oxygenation function are easily applicable at the patient’s bedside and can significantly improve oxygenation [14]. The size of cannulas plays a crucial role in achieving higher ECMO blood flow rates, but unfortunately, this parameter is not always modifiable after implantation. Alternate ECMO configurations, such as additional venous cannula, a veno-pulmonary arterial configuration to lower recirculation, or a veno-venous pulmonary arterial (V-VP) ECMO configuration (combining a femoral drainage cannula and a veno-pulmonary cannula for arterial return), could be proposed as options to further address refractory hypoxemia [15].

Our study has several limitations. First, it is monocentric and retrospective with a small sample size. Second, timing of hemodynamic evaluation was not protocolized resulting in time differences between the two assessments. Third, several pharmacological interventions were carried out on the same patients, which could bias the results.

## Conclusions

Our protocol for refractory hypoxemia under VV-ECMO using ivabradine and beta-blockers had a good immediate tolerance and resulted in a better ECMO flow/CO ratio and arterial saturation but with a decreased DO_2_. Further research is warranted to explore and refine the management strategies for refractory hypoxemia during VV-ECMO.

## Supplementary Information

Below is the link to the electronic supplementary material.

Supplementary file1 (DOCX 75 KB)

Supplementary file2 (DOCX 24 KB)

## Data Availability

The dataset used during the current study is available from the corresponding author upon reasonable request.

## Abbreviations

ARDS: Acute respiratory distress syndrome
CI: Cardiac index
CO: Cardiac output
DO_2_: Oxygen delivery
ICU: Intensive care unit
MAP: Mean arterial pressure
SaO2: Arterial oxygen saturation
TACO: Transfusion-associated circulatory overload
TRALI: Transfusion-related acute lung injury
VV-ECMO: Venovenous extracorporeal membrane oxygenation
V-VP ECMO: Veno-venous pulmonary arterial extracorporeal membrane oxygenation
