# Supplementary material for: Control of Cardiac Output with Ivabradine or Beta-Blockers for Refractory Hypoxemia under Veno-Venous ECMO for Severe ARDS
Source: Cardiovasc Drugs Ther. 2024 Dec 30;39(5):961–6. doi: 10.1007/s10557-024-07650-5 (PMC12602563; doi:10.1007/s10557-024-07650-5)

## Figure S1: Flow chart. * One patient did not receive ivabradine and was directly treated with beta-blockers because the enteral route was not available


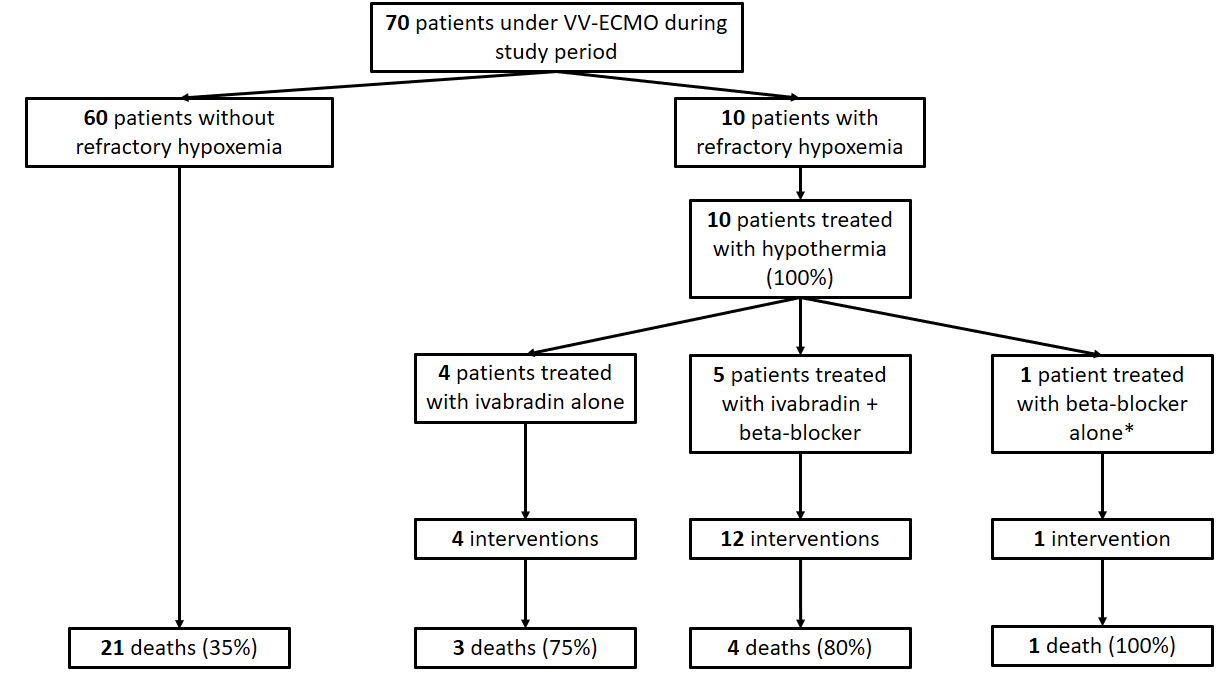

Supplement: Supplementary file 1 — Supplementary file1 (DOCX 75 KB) [file 10557_2024_7650_MOESM1_ESM.docx]
