# Supplementary material for: Control of Cardiac Output with Ivabradine or Beta-Blockers for Refractory Hypoxemia under Veno-Venous ECMO for Severe ARDS
Source: Cardiovasc Drugs Ther. 2024 Dec 30;39(5):961–6. doi: 10.1007/s10557-024-07650-5 (PMC12602563; doi:10.1007/s10557-024-07650-5)
Supplement: Supplementary file 2 — Supplementary file2 (DOCX 24 KB) [file 10557_2024_7650_MOESM2_ESM.docx]

## Table S1: Patients characteristics and outcome in ICU

| **Variables** | n=10 |
| --- | --- |
| Age, years | 48 [33-58] |
| Male gender | 6 (60) |
| Body mass index, kg/m² | 32 [29-35] |
| **Comorbidities** |  |
| Diabetes mellitus | 1 (10) |
| Arterial hypertension | 4 (40) |
| Ischemic heart disease | 1 (10) |
| Heart failure | 0 (0) |
| COPD | 0 (0) |
| **Delays** |  |
| From ECMO implantation to ivabradine introduction, days | 13 [7-21] |
| From ivabradine end to ECMO explantation or death, days | 45 [2.5-88] |
| Duration of ivabradine treatment, days | 16 [1.5-19] |
| From end of ivabradine to death, days | 16 [2-45] |
| From ECMO implantation to beta blocker introduction, days | 19 [11-34] |
| From beta blocker end to ECMO explantation or death, days | 22 [1-68] |
| Duration of beta blocker treatment, days | 7 [1-29] |
| From end of beta blocker to death, days | 10 [1-39] |
| **Outcomes** |  |
| VAP | 10 (100) |
| ICU length of stay, days | 81 [37-120] |
| Renal replacement therapy | 7 (70) |
| ECMO duration, days | 59 [36-92] |
| Death in hospital | 8 (80) |
| Cause of death:   - MOF - Intracranial bleeding - Haemorrhagic shock - Withholding or withdrawing of life-sustaining treatments | 7 (70)  2 (20)  3 (30)  3 (30) |
| **Data are median [interquartile range] or number (percentage). ICU: intensive care unit; COPD: chronic obstructive pulmonary disease, ECMO: extracorporeal membrane oxygenation; VAP: ventilator associated pneumonia; MOF: multiple organ failure.** | |

## Table S2: Patient management (n=10) before pharmacological intervention (n=17) for refractory hypoxemia

|  | **Pharmacological intervention**  **(n=17)** | **Ivabradin**  **(n=9)** | **Beta blocker (n=8)** |
| --- | --- | --- | --- |
| **Biology before pharmacological intervention**^$^ | | | |
| Lacticodeshydrogenase (U/L) | 719 [518-925] | 638 [493-1010] | 778 [628-898] |
| Platelet (G/L) | 128 [95-152] | 128 [95-152] | 123 [80-153] |
| Free hemoglobin plasmatic (mg/L) | 110 [52-202] | 157 [36-272] | 94 [66-112] |
| Fibrinogen (g/L) | 5.9 [2.9-6.5] | 6.4 [2.6-6.5] | 5.5 [4-6.5] |
| Carboxy hemoglobin (%) | 2.9 [1.6-3.4] | 2.1 [1.6-3.4] | 3.2 [1.8-3.4] |
| **Transfusions and bleeding before pharmacological intervention*** | | | |
| Packed Red blood cells* |  |  |  |
| Patients transfused | 15 (88) | 8 (89) | 7 (88) |
| Units received (in transfused patients) | 2 [1-3] | 2 [1.5-4] | 1.5 [1-2.8] |
| Platelets concentrates* |  |  |  |
| Patients transfused | 2 (12) | 1 (11) | 1 (13) |
| Bleeding^$^ | 13 (76) | 7 (78) | 6 (75) |
| **Other interventions** |  |  |  |
| Prone position^$^ | 9 (53) | 3 (33) | 6 (75) |
| Membrane change* | 5 (29) | 4 (44) | 1 (13) |
| ***within 72 hours; $within 48 hours** | | | |
